# Patellofemoral pain syndrome

**DOI:** 10.1007/s00167-013-2759-6

**Published:** 2013-11-13

**Authors:** Wolf Petersen, Andree Ellermann, Andreas Gösele-Koppenburg, Raymond Best, Ingo Volker Rembitzki, Gerd-Peter Brüggemann, Christian Liebau

**Affiliations:** 1 Klinik für Orthopädie und Unfallchirurgie, Martin Luther Krankenhaus, Berlin, Grunewald, Caspar Theyss Strasse 27-31, 14193 Berlin, Germany; 2 Arcus Sportklinik Pforzheim, Pforzheim, Germany; 3 Asklepios Harzkliniken GmbH Fritz-König-Stift, Bad Harzburg, Germany; 4 Deutsche Sporthochschule Köln, Institut für Biomechanik, Cologne, Germany; 5 Sportklinik Stuttgart, Stuttgart, Germany; 6 Cross Klinik Basel, Olympic Medical Center, Basel, Switzerland

**Keywords:** Functional malalignment, Dynamic valgus, Hip strength, Rear-foot eversion, Single-leg squat, Hip strength

## Abstract

The patellofemoral pain syndrome (PFPS) is a possible cause for anterior knee pain, which predominantly affects young female patients without any structural changes such as increased Q-angle or significant chondral damage. This literature review has shown that PFPS development is probably multifactorial with various functional disorders of the lower extremity. Biomechanical studies described patellar maltracking and dynamic valgus in PFPS patients (functional malalignment). Causes for the dynamic valgus may be decreased strength of the hip abductors or abnormal rear-foot eversion with pes pronatus valgus. PFPS is further associated with vastus medialis/vastus lateralis dysbalance, hamstring tightness or iliotibial tract tightness. The literature provides evidence for a multimodal non-operative therapy concept with short-term use of NSAIDs, short-term use of a medially directed tape and exercise programmes with the inclusion of the lower extremity, and hip and trunk muscles. There is also evidence for the use of patellar braces and foot orthosis. A randomized controlled trial has shown that arthroscopy is not the treatment of choice for treatment of PFPS without any structural changes. Patients with anterior knee pain have to be examined carefully with regard to functional causes for a PFPS. The treatment of PFPS patients is non-operative and should address the functional causes.

*Level of evidence* V.

## Introduction

The incidence of “anterior knee pain” is high and is located at 22/1,000 persons per year [12, 60]. Women are affected about more than twice as often as men [12, 29, 60]. The causes for anterior knee pain are multifactorial. These include overuse injuries of the extensor apparatus (tendonitis, insertional tendinosis), patellar instability, chondral and osteochondral damage [52].

The patellofemoral pain syndrome (PFPS) is a common cause for “anterior knee pain” and mainly affects young women without any structural changes such as increased Q-angle or significant pathological changes in articular cartilage [1, 12, 29, 52, 60]. Therefore, PFPS is a diagnosis of exclusion [1].

Other associated manifestations include crepitus and functional deficit [1]. PFPS symptoms cause many athletes to limit their sportive activities [10]. According to some authors, the PFPS will eventually lead to osteoarthritis [45, 64, 67].

The pathogenesis of PFPS is multifactorial with various functional disorders of the lower extremity to be involved [8].

Aim of this literature review is to summarize evidence regarding the underlying pathology of PFPS and the best way to treat this condition.

### “Patella tracking” in patient with PFPS

The role of the patella maltracking for the emergence of the PFPS has long been a controversial issue.

Recent studies, however, show that maltracking of the patella probably plays a key role. Draper et al. [24] for example have demonstrated by dynamic MRI that patients with a PFPS squat with increased lateralization and increased lateral tilt of the patella. Witvrouw et al. [74] showed that a hypermobile patella had a significant correlation with the incidence of patellofemoral pain.

**Fig. 1:**
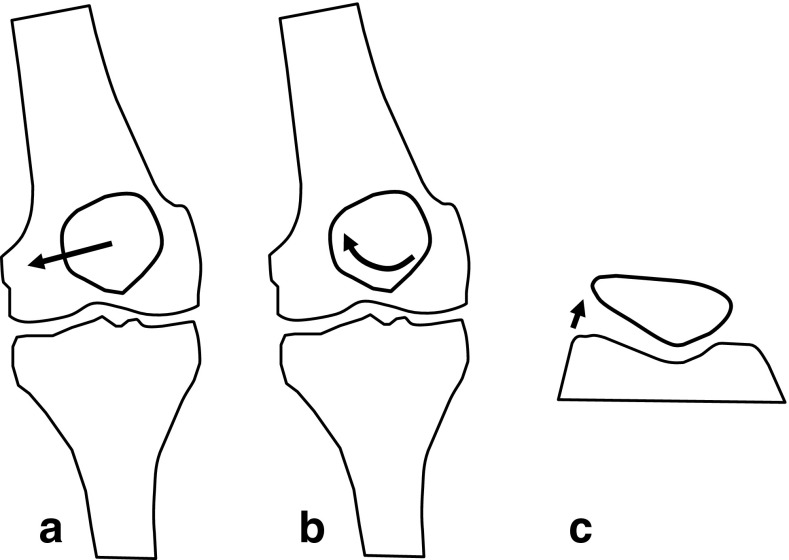
This schematic drawing shows results of an experimental study published by Wilson et al. [72]. Patients with PFPS had significantly increased lateral shift (**a**), lateral spin (**b**) and a tendency towards increased lateral tilt (**c**)

Wilson et al. [72] used skin marker and an optoelectronic motion capture system to examine gliding of the patella in patients with a PFPS in a standing position and while squatting. In this study, the patella of patients with PFPS had significantly increased lateral translation (maltracking), lateral patellar spin and a tendency towards increased lateral tilt compared to healthy subjects [72] (Fig. 1).

### Role of M. vastus lateralis and M. vastus medialis

Pal et al. [50] have demonstrated that “patella maltracking” in patients with PFPS correlates with a delayed activation of the M. vastus medialis. An imbalance in the activation of the M. vastus medialis obliquus and M. vastus lateralis was also shown by Cowan et al. [19]. In patients with a PFPS, the M. vastus lateralis was earlier activated than the M. vastus medialis obliquus when patients climbed downstairs and upstairs. In the control group that imbalance did not exist. These findings were supported by several other studies [15, 16, 74]. Patients with patellofemoral problems exhibited atrophy of the vastus medialis obliquus [49].

Despite these results, however, it is not clear whether the M. vastus lateralis and medialis imbalance are the primary cause for patellar maltracking.

### Static or dynamic malalignment?

The role of the Q-angle (static measure) as predictor for PFPS is discussed controversially [39, 41, 45, 51, 59].

Some authors report that an increased Q-angle is associated with PFPS [39, 41]. For example, Rauh et al. [59] found that cross-country runners with increased Q-angle (>20°) are more prone to knee injury than athletes with normal Q-angle.

In contrast, Park et al. [51] have shown that the Q-angle is not increased in PFPS patients. Other reports also do not show strong correlations between static measures such as the Q-angle to the onset of PFPS [51].

**Fig. 2:**
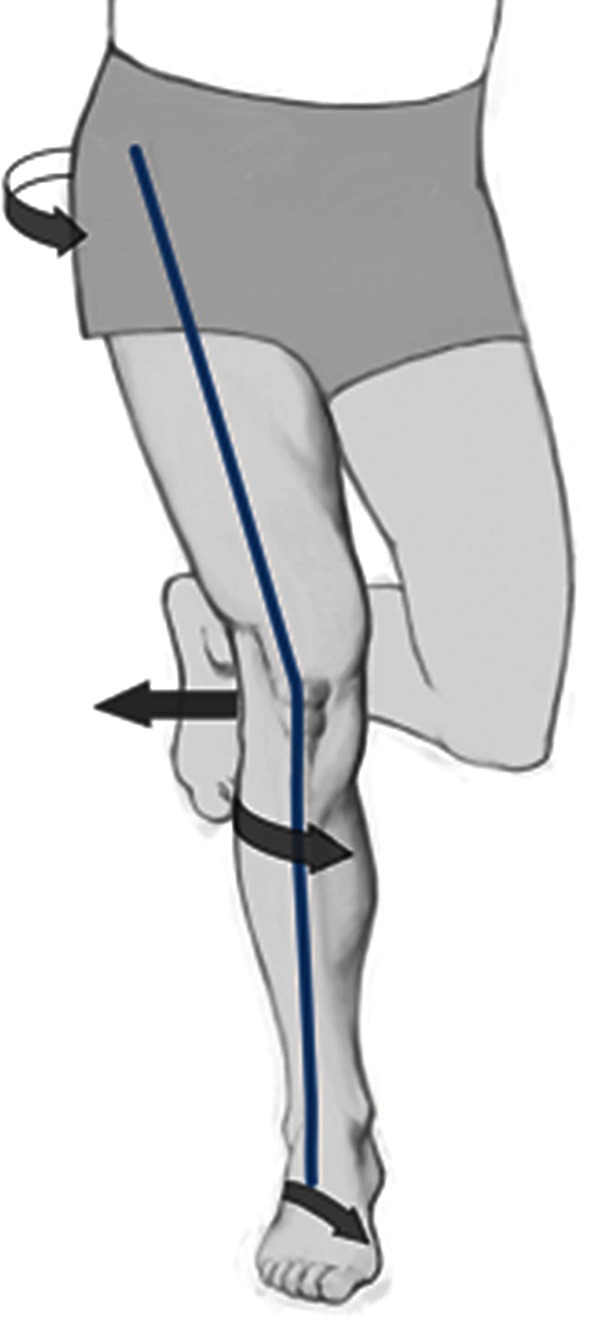
Cause for functional or dynamic valgus can be internal rotation of the femur, the tibia or both. Internal rotation of the femur might be the result of weakness of the hip abductors; internal rotation of the tibia might arise from rear-foot eversion or pes pronatus. Functional valgus may lead to lateral patella maltracking

That means that the cause for maltracking of the patella and the imbalance of the vastus medialis and lateralis in some patients with a PFPS may not be part of a structural fault (Fig. 2).

Rather a dynamic or functional malalignment is seen in these patients [21, 52].

Myer et al. [45] studied female middle and high school basketball players. In this study, athletes who developed a new PFPS demonstrated increased knee abduction moments of the symptomatic limb. That means there is a dynamic valgus position of the knee joint, which might be reinforced by an internal rotation of the femur and tibia (Fig. 2).

A dynamic valgus alignment is more frequently observed in female athletes compared to males [27, 28]. These biomechanical and neuromuscular mechanisms may be links to the pathogenesis of PFPS in young female athletes [35, 52, 56]. The functional or dynamic valgus may influence patella tracking leading to lateralization of the patella [43]. Souza et al. [63] performed kinematic imaging of the patellofemoral joint using an open MRI to measure femur and patella rotation in PFPS patients. In this study, altered patellofemoral joint kinematics in females with PFPS was related to excessive medial rotation of the femur and lateral rotation of the patella [63].

**Fig. 3:**
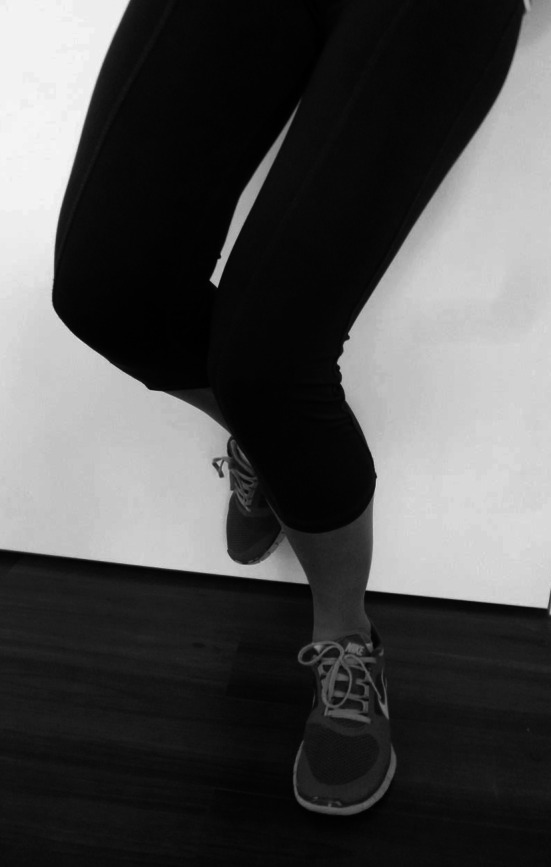
Dynamic valgus can be visualized by one-legged squats. Young female athlete with dynamic valgus and PFPS

The functional “malalignment” or dynamic valgus can be visualized clinically with one-legged squats (Fig. 3). Crossley et al. [21] have demonstrated that a valgus collapse of the knee joint during one-legged squat indicates weakness of the hip abductors.

### Hip stability and hip abductor strength

Recent research has shown that functional malalignment does not arise in the knee joint but rather by internal rotation of the femur due to weakness of hip external rotators and abductors (M. gluteus medius and minimus) [2, 13, 14, 46, 57].

Padua et al. [46] found that decreased M. gluteus medius and M. gluteus maximus strength is related to increased knee valgus after landing a drop jump. Brent et al. [14] showed that females have decreased relative hip abduction strength in comparison with males. Decreased relative hip abduction strength has also been demonstrated in patients with PFPS [2, 13]. Bolgla et al. [13] showed that patients with a PFPS have a significant weakness of the external rotators of the hip. Baldon et al. [2] could also demonstrate that patients with PFPS have lower hip abduction strength.

All these studies are supported by a systematic review which demonstrated strong evidence that females with PFPS have a decreased hip abduction, external rotation and extension strength compared with healthy controls [57]. This evidence does not exist for male PFPS patients.

**Fig. 4:**
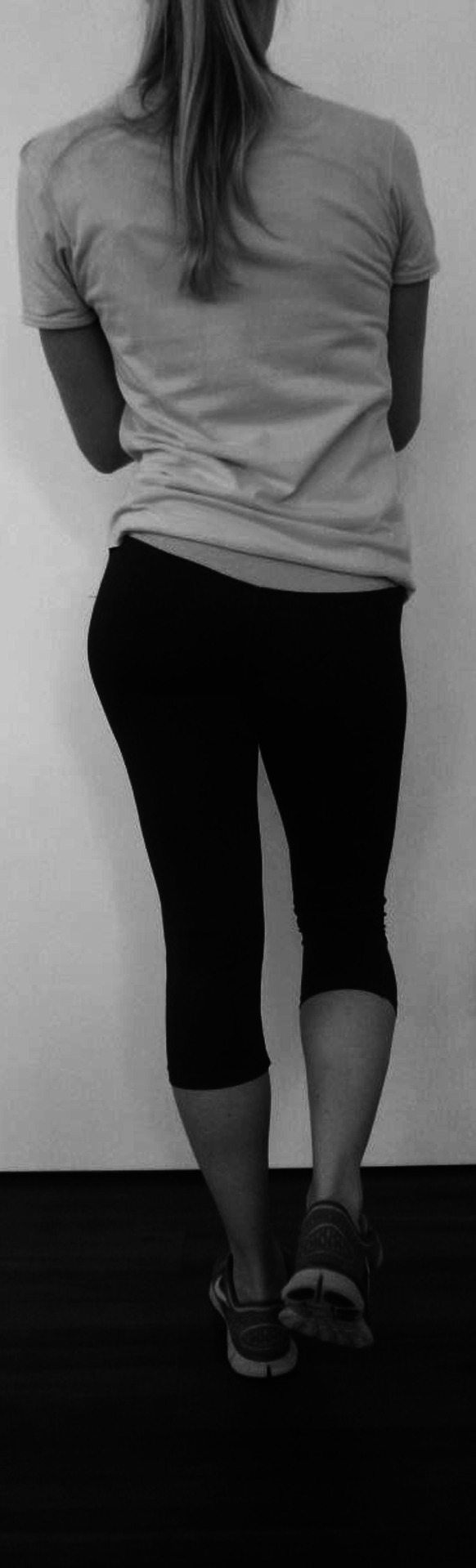
Hip muscle weakness can be demonstrated by having the patient raise the contralateral leg. If the patient’s hip cannot keep up 1 min and hip drops, then that is a sign of a weak abductor

Petersen et al. [52] have shown that the stability of the pelvis in PFPS patients can be checked clinically with the patient standing on one leg (Fig. 4). If the patient cannot stabilize the pelvis for 1-min standing on the affected leg, this is a sign of weakness of the hip muscles [52].

### Rear-foot eversion

An internal rotation of the tibia can also be caused by rear-foot eversion [42, 52] (Figs. 2, 3).

A systematic review published by Barton et al. [4] has shown that patients with PFPS may have several disorders of the foot mechanics. These disorders include delayed timing of peak rear-foot eversion, increased rear-foot eversion at heel strike and reduced rear-foot eversion range [4].

**Fig. 5:**
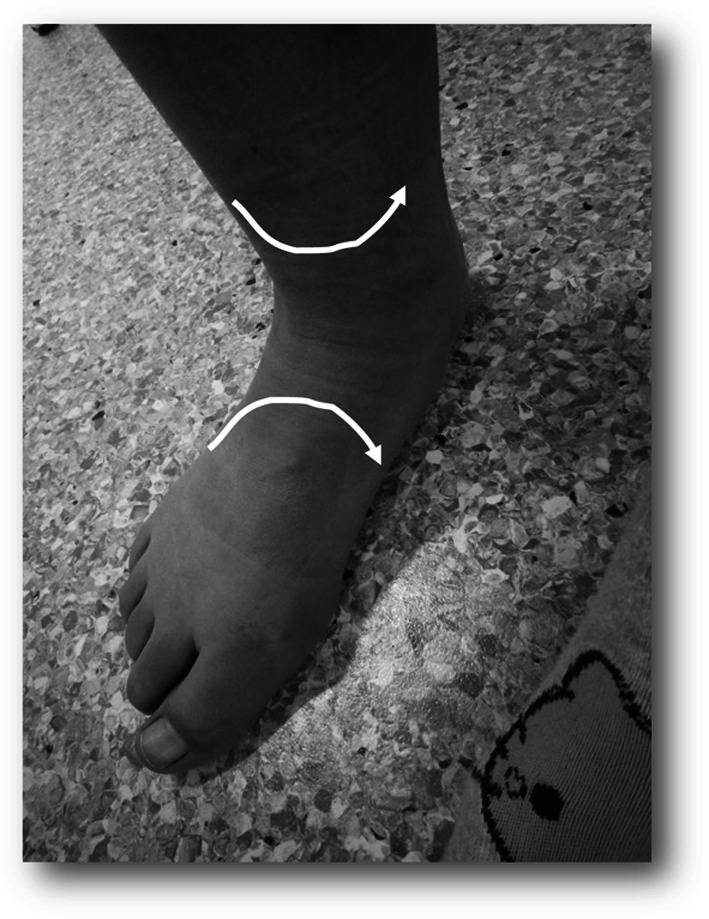
Patient with the combination of pes pronatus valgus and PFPS. In this patient, internal rotation of the foot leads to internal rotation of the tibia

In a recent study, these authors could show [5] a relationship between earlier rear-foot eversion and the emergence of PFPS. In another study, Barton et al. [3] could also show that increased peak rear-foot eversion was associated with increased peak internal rotation of the tibia in PFPS patients (Fig. 5).

Mølsgaard et al. [44] also could demonstrate abnormalities of the navicular bone in high school students with PFPS such as increased navicular drop, navicular drift and dorsiflexion.

Barton et al. [6] showed that PFPS patients have a more pronated foot type, increased forefoot abduction and increased rear-foot eversion in comparison with a healthy control group.

In conclusion, the literature provides evidence for rear-foot and forefoot abnormalities in PFPS patients.

### Iliotibial tract

Dynamic valgus may also have influence on the length of the iliotibial tract. Wu et al. [76] have shown that the iliotibial tract may also have an influence on patellar tracking. This may be anatomically explained by Kaplans fibres which connect the iliotibial tract with the patella. Other studies about the role of the iliotibial in PFPS patients are lacking.

### Hamstring imbalance and tightness

There is some evidence in the literature that there is not only abnormal frontal plane knee motion in patients with PFPS.

Two studies identified a significant association between PFPS and hamstring tightness [47, 71]. These authors found significant hamstring tightness in patients relative to the control group. In another study, Patil et al. [48] showed by EMG that in patients with PFPS, the lateral hamstrings contracted earlier than the medial hamstrings during maximal arbitrary isometric contractions.

Besier et al. [9] have shown that patients with PFPS have greater co-contraction of the quadriceps and hamstrings compared to controls without symptoms of PFPS. In this study, females showed 30–50 % higher hamstring and gastrocnemius muscle forces during both walking and running compared to males. These authors concluded that by this mechanism, some PFPS patients might experience increased joint contact force and joint stress in comparison with healthy subjects.

All these changes can lead to high stress on the patella and its supporting structures.

### “Knee-spine syndrome” [66]

Tsuji et al. [66] examined the correlation between patellofemoral joint pain, lumbar lordosis and sacral inclination, in elderly patients with anterior knee pain. There was a significant difference in sacral inclination between subjects with and without anterior knee pain [66]. Inclination of the sacrum was less (app 5°) in patients with patellofemoral pain [66]. This pathological concept was called the “knee-spine syndrome”.

For younger patients with PFPS, however, this mechanism has not been examined. More research is needed to elucidate the role of this mechanism for the pathogenesis of PFPS.

### Psychological factors contributing to PFPS

The importance of psychological factors for the development of PFPS should not be underestimated [36, 37, 54, 55, 65].

Jensen et al. [37] have shown that pain and knee function can also be associated with psychological factors in some patients with long-lasting PFPS. These authors have shown that patients with PFPS have higher levels of mental distress in comparison with healthy subjects.

Thomee et al. [65] found that there are similarities regarding the pain experience and pain coping of PFPS patients to other groups of patients with chronic pain. In contrast to other chronic pain patients, higher scores were found on the Pain Catastrophizing Scale [65].

Piva et al. [36, 37] have identified a psychological predictor for pain and function in PFPS patients. This predictor was fear-avoidance belief about physical activity.

Domenech et al. [23] support the fear-avoidance model. This study demonstrated a high incidence of psychological distress such as anxiety and depression, pain catastrophizing and kinesophobia in PFPS patients. These factors were strong predictors of pain and disability in PFPS patients.

In some cases, the knee problems can be triggered by secondary disease profit [52]. This may play a role in young competitive athletes, who are no longer capable of the increasing demands. The knee problems then may serve as an explanation for the stagnation or reduced performance [52].

### Trigger for the PFPS

**Fig. 6:**
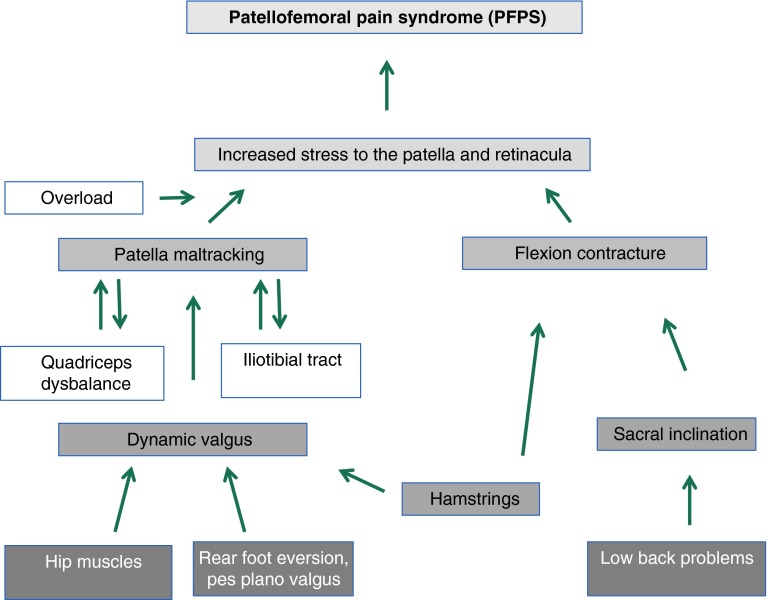
This algorithm summarizes the findings from the literature, which explains the pathogenesis for PFPS

Figure 6 summarizes a possible pathogenesis for the patellofemoral pain syndrome based on findings from the literature.

A possible trigger for the patellofemoral pain syndrome may be overload of the patellofemoral joint (e.g. high-intensity training). The combination of overload with dynamic valgus and functional lateralization of the patella may lead to overuse of the structures of the patellofemoral joint [43]. This overload may cause anterior knee pain.

Anterior knee pain can be a vicious cycle. By anterior knee pain, the muscle activity of the lower extremity may be further inhibited.

### Neurophysiological cause of pain in patients with PFPS

The exact cause of pain in patients with PFPS is unclear. Most probably the pain develops in the insertions of the extensor mechanism or within the subchondral bone [26].

Woitys et al. [75] observed substance P rich free nerve endings within the retinacula, Hoffa’s fad pad and subchondral bone.

Sanchis-Alfonso and Roselló-Sastre [61] observed a high expression of several neural markers such as neurofilament protein, S-100 protein, neural growth factor and substance P in the lateral retinacula of patients with patellofemoral maltracking. This study demonstrates that the innervation of the retinacula may play a role for the development of anterior knee pain. Sanchis-Alfonso [62] hypothesized that Sustance P and NGF expression may be related to mechanical stress within the retinacula. Patellar maltracking might be the cause for this abnormal stress [62].

In an experimental arthroscopy with local skin anaesthesia, S. Dye experienced strong pain when then probe touched the retinacula, Hoffas fad pad or the peripatella synovium [26].

However, there is additional evidence that the subchondral bone may also play a role for the origin of pain in PFPS patients. Draper et al. [24] have demonstrated increased metabolic bone activity in patients with PFPS using F NaF PET/CT.

Rathleff et al. [58] have shown that not only peripheral but also central mechanisms may cause pain in patients with PFPS. At the knee, adolescents with PFPS had a significantly lower pressure pain thresholds (localized hyperalgesia) compared with controls. On the tibialis anterior, however, adolescents with PFPS had also a lower pressure pain thresholds (distal hyperalgesia) compared with controls. Jensen et al. [36] could demonstrate an aberrant sensory function in PFPS patients. The mean detection threshold for temperature was significantly increased in PFPS patients compared to healthy controls.

## Therapy

### Surgical versus conservative therapy

Kettunen et al. [40] have demonstrated in a prospective randomized study with PFPS patients that an arthroscopy, in combination with physiotherapy, had no positive effect compared to physiotherapy alone. Therefore, the treatment of PFPS is primarily non-operative.

### Pharmacological therapy

A meta-analysis has shown that there is limited evidence for the effectiveness of non-steroidal anti-inflammatory drugs for reduction in acute anterior knee pain in PFPS patients [34]. The findings regarding the efficacy of glycosamino-glycanpolysulfate and intraarticular application of corticoids are contradictory [34].

### Tape

**Fig. 7:**
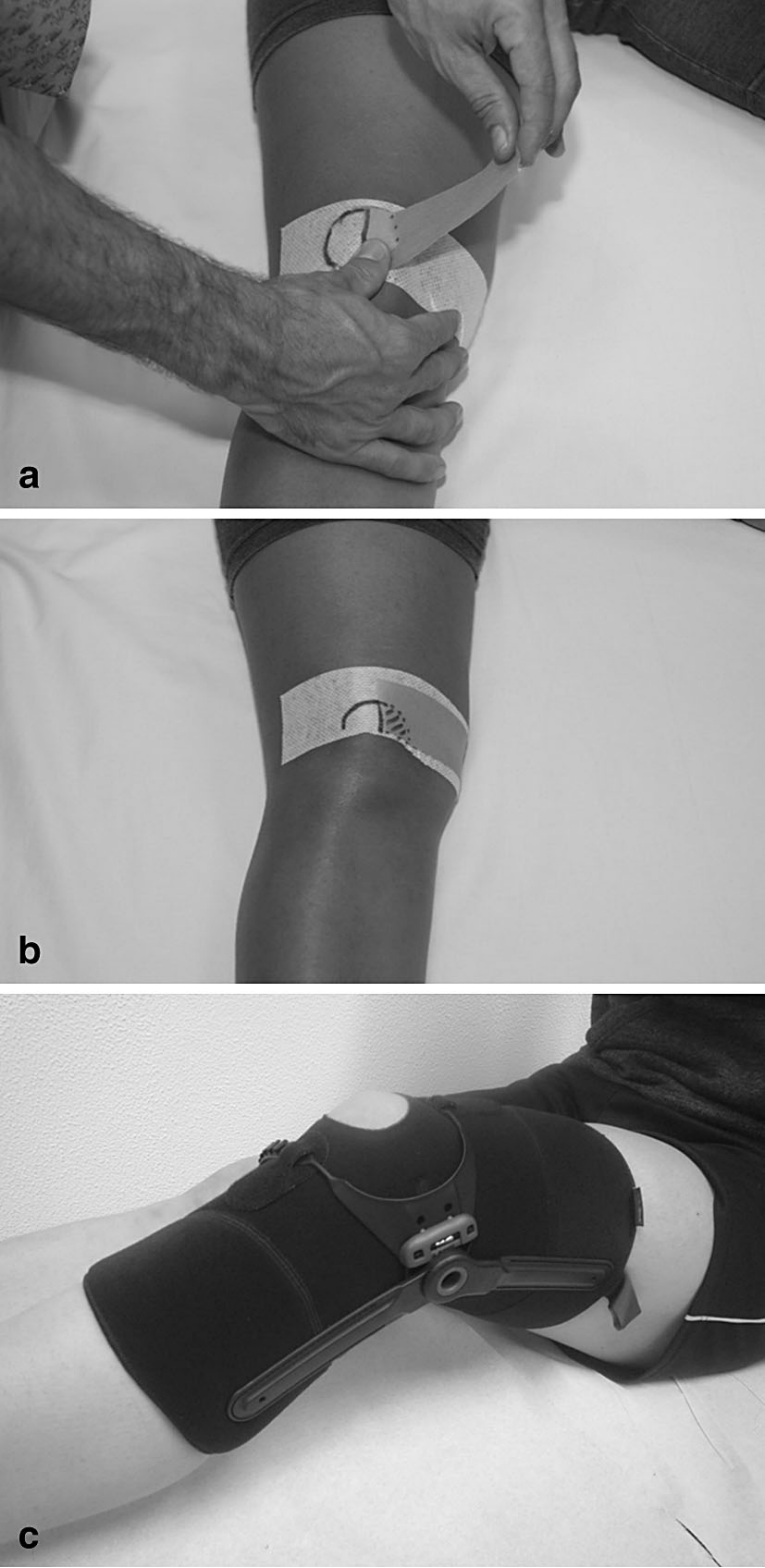
**a**, **b** Application of a classical Mc Connel tape, **c** Example of a patella brace (Patella pro) which can apply a medially directed force to the patella

Aim of classical taping is to modify patella tracking by applying adhesive tape stripes to the skin (Fig. 7). The tape should apply a medially directed force to counteract lateral patella maltracking. The most popular application is the Mc Connel tape.

Pfeiffer et al. [53] demonstrated that a medially directed tape has the potential to correct lateral patella maltracking and patellar tilt. Gilleard et al. [30] showed that the M. vastus medialis obliquus (VMO) is activated earlier by application of a medially directed tape. Similar results were reported by Cowan et al. [20]. According to Christou et al. [17], patients with patellofemoral pain syndrome had a higher VMO activity when a medially directed tape was applied.

Clinical studies could support these findings and showed that traditional medially directed tape has a positive effect on the symptoms of PFPS [22, 70]. Two meta-analyses were published evaluating the effect of tape on pain reduction in PFPS patients [22, 70]. One meta-analysis was published in 2002 and found that adhesive tape combined with exercise was significantly superior to an exercise programme alone regarding functional improvement and decrease in pain [22].

Another meta-analysis was published by Warden et al. [70] in 2008. This study showed that medially directed tape produces a clinically meaningful reduction in knee pain in patients with PFPS. This study, however, has also shown that even sham tape application has a positive effect on pain reduction in PFPS patients. Therefore, placebo, proprioceptive or sensory skin effects may contribute to the beneficial tape effects. The data analysis, however, revealed that these effects only explain approximately half of the pain reduction associated with medially directed tape only [70].

However, it should be noted that the tape effect on pain reduction has only been investigated in short-term studies (12-week follow-up). Long-term effects of tape on anterior knee pain have not been established. Therefore, the current evidence supports the use of tape as a temporary pain-relieving treatment of anterior knee pain in PFPS patients [70].

The positive influence of the tape on pain and function probably explains the synergistic effect of tape and physiotherapy. The simultaneous application of a restraining tape and a physiotherapy exercise programme achieved a better role as the sole tape system [22].

### Patella braces

Patella braces are non-adhesive devices also applying an external medially directed force which may counteract lateral patella maltracking (Fig. 7).

Draper et al. [25] have demonstrated by real-time MRI that a knee brace which applies a medially directed force on the patella can reduce the lateralization of the patella and tilt in women with PFPS significantly better than a bandage. Powers et al. [56] analysed an orthosis which applied a medially directed force on the patella in PFPS patients. Theses authors found that pain was decreased and quadriceps activation was promoted.

According to the meta-analysis published by D’hondt et al. [22], the use of a patella brace has positive effects on pain, function (Kuala score) and the patellofemoral congruence angle as compared to a control group without the treatment. Due to the low quality of the studies, the authors concluded that this evidence should be regarded as limited.

Warden et al. [70] also found disputable evidence for the use of orthotics for PFPS patients. In this meta-analysis, only one of three studies found an effect of a medially directed patella brace, whereas in two studies, the effect was not significant. In these studies, no difference was found difference between orthoses which can apply a medially directed force on the patella and sham orthoses.

Therefore, we conclude that better designed studies should be performed to evaluate the effect of braces on pain and function in patients with PFPS.

### Foot orthotics

Increased rear-foot eversion and pes pronatus can favour internal rotation and thus a dynamic valgus position of the lower extremity [3–5, 11, 44]. Therefore, insoles or foot orthotics could be a treatment option to correct the malalignment.

We found no meta-analysis analysing the effect of insoles on pain in PFPS patients and the studies from the literature report conflicting results.

Wiener-Ogilvie et al. [73] randomized PFPS patients to three different groups. These groups received either a foot orthoses alone, physiotherapy alone, or a combination of physiotherapy and foot orthoses. After a short-term follow-up, these authors found no significant difference in outcome among the three groups regarding pain levels or function.

In a prospective study, the Western Ontario and McMaster University Osteoarthritis Index (WOMAC) significantly increased by the administration of a foot orthosis in patients with the combination of pes plano valgus, and patellofemoral pain syndrome after 2 weeks and 3 months [31].

Vicenzo et al. [68] published a prospective randomized trial involving 179 patients with a PFPS. Participating patients were divided into four different treatment groups: (1) foot orthosis, (2) a flat insert, (3) physiotherapy or (4) physiotherapy and a foot orthosis. Improvement for pain and function was found in all groups at 52 weeks, but there was no significant difference between the g treatment groups. In a post hoc subgroup analysis of the patients who received a foot orthosis, these authors found some predictors for the efficacy of foot orthoses in PFPS patients [69]. These positive predictors were body height less than 165 cm, age older than 25, lower pain levels and midfoot abnormalities [69].

These findings may explain why other studies found an effect of foot orthosis on pain in PFPS patients. These studies, however, included patients with the combination of pes plano valgus and PFPS [7–9, 18].

Collins et al. [18] published a prospective randomized study involving 179 patients with PFPS and pes pronatus. These authors found that foot orthotics had the same effect as physical therapy. However, a synergistic effect of both therapies did not exist.

Barton et al. [7] examined the effect of prefabricated foot orthotics in patients with PFPS and pes plano valgus in a cohort study involving 60 patients. These authors could demonstrate that the use of a foot orthotic leads to pain reduction and improvements in functional parameters such as leg raise numbers and step down numbers. In another study, Barton et al. [8] could show that greater rear-foot eversion is a predictor for the efficacy of foot orthotics in individuals with PFPS. In another study, the quality of life of patients with the combination of PFPS and pes plano valgus could be improved through the use of foot orthotics [38].

Therefore, foot orthosis might be a treatment option for patients with the combination of disorders of foot posture and PFPS. However, more well-designed studies are needed to examine the efficacy of foot orthoses and to identify subgroup patients which would benefit from the treatment with foot orthoses.

### Physical rehabilitation

Physiotherapy is the most frequently studied form of therapy for PFPS [33]. Two meta-analyses have been published.

In 2003, Heintjes et al. [33] published a Cochrane review about exercise for PFPS. This meta-analysis identified one high- and two low-quality studies comparing exercise with a control group without exercise. This meta-analysis reported the positive effects for pain reduction for patients treated with exercise. One of the low-quality studies even reported also functional improvement in the exercise groups.

In a more recent meta-analysis published in 2008, 10 prospective randomized studies could be analysed [32]. All these studies showed a positive effect of exercise on pain reduction. Positive results have been described in particular with active stretching exercises, squats, ergometer, static quadriceps exercises, active leg raises, leg press, raising and lowering climbing exercises. Four of the exercise programme also included exercises to strengthen the hip abductors. In one study, trunk-stabilizing exercises, including the rectus abdominis, were analysed. The most frequent duration of the exercise programmes was 6 weeks. The exercises were conducted two-to-four times daily with 10 repetitions. Closed- and open-chain exercises, balance training and proprioceptive exercises have been described. Eight studies also included stretching exercises. In 8 studies, additional interventions such as restriction of symptom inducing activities, tape, braces and NSAIDs were allowed.

**Fig. 8:**
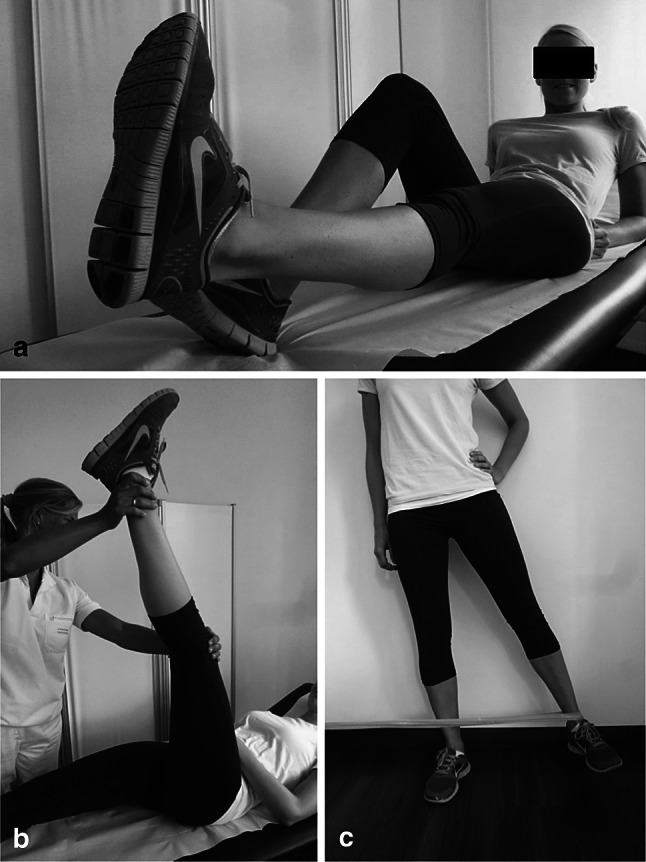
Exercises for the treatment of PFPS should include **a** the quadriceps, **b** the hamstrings and **c** the hip muscles

In summary, there is strong evidence for exercise in the treatment of PFPF in the literature. These exercises should address hip muscles, trunk stability, quadriceps, hamstrings and the iliotibial tract (Fig. 8).

## Conclusions and future strategies

The patellofemoral pain syndrome is a clinical entity which leads to anterior knee pain in patients without any pathological changes at the cartilage of the patellofemoral joint. Patellar maltracking due to a functional malalignment or dynamic valgus may be an underlying cause for this clinical entity. Possible causes for the dynamic valgus may be decreased strength of the hip abductors or a pes pronatus valgus. The functional malalignment is associated with quadriceps dysbalance, hamstring tightness or iliotibial tract tightness. The clinical usefulness of these findings is that a physician may tailor an individual treatment plan for each patient according to the underlying pathology. However, there is a clear need for further research to distinguish between cause and effect.

The literature provides evidence for a multimodal non-operative therapy concept with short-term use of NSAIDs, short-term use of a medially directed tape and complex exercise programmes with the inclusion of the lower extremity, and hip and trunk muscles. There is also evidence for the use of patellar braces and foot orthosis. There is need for further studies about braces and foot orthosis.

## References

[1] Al-Hakim W, Kumar Jaiswal P, Khan W, Johnstone D (2012). The non-operative treatment of anterior knee pain. Open Orthop J.

[2] Baldon R, Nakagawa TH, Muniz TB, Amorim CF, Maciel CD, Serrão FV (2009). Eccentric hip muscle function in females with and without patellofemoral pain syndrome. J Athl Train.

[3] Barton CJ, Levinger P, Crossley KM, Webster KE, Menz HB (2012). The relationship between rearfoot, tibial and hip kinematics in individuals with patellofemoral pain syndrome. Clin Biomech (Bristol, Avon).

[4] Barton CJ, Levinger P, Menz HB, Webster KE (2009). Kinematic gait characteristics associated with patellofemoral pain syndrome: a systematic review. Gait Posture.

[5] Barton CJ, Levinger P, Webster KE, Menz HB (2011). Walking kinematics in individuals with patellofemoral pain syndrome: a case-control study. Gait Posture.

[6] Barton CJ, Bonanno D, Levinger P, Menz HB (2010). Foot and ankle characteristics in patellofemoral pain syndrome: a case control and reliability study. J Orthop Sports Phys Ther.

[7] Barton CJ, Menz HB, Crossley KM (2011). Effects of prefabricated foot orthoses on pain and function in patients with patellofemoral pain syndrome: a cohort study. Phys Ther Sport.

[8] Barton CJ, Menz HB, Levinger P, Webster KE, Crossley KM (2011). Greater peak rearfoot eversion predicts foot orthoses efficacy in individuals with patellofemoral pain syndrome. Br J Sports Med.

[9] Besier TF, Fredericson M, Gold GE, Beaupré GS, Delp SL (2009). Knee muscle forces during walking and running in patellofemoral pain patients and pain-free controls. J Biomech.

[10] Blond L, Hansen L (1998). Patellofemoral pain syndrome in athletes: a 5.7-year retrospective follow-up study of 250 athletes. Acta Orthop Belg.

[11] Boldt AR, Willson JD, Barrios JA, Kernozek TW (2013). Effects of medially wedged foot orthoses on knee and hip joint running mechanics in females with and without patellofemoral pain syndrome. J Appl Biomech.

[12] Boling M, Padua D, Marshall S, Guskiewicz K, Pyne S, Beutler A (2010). Gender differences in the incidence and prevalence of patellofemoral pain syndrome. Scand J Med Sci Sports.

[13] Bolgla LA, Malone TR, Umberger BR, Uhl TL (2008). Hip strength and hip and knee kinematics during stair descent in females with and without patellofemoral pain syndrome. J Orthop Sports Phys Ther.

[14] Brent JL, Myer GD, Ford KR, Hewett TE (2008). A longitudinal examination of hip abduction strength in adolescent males and females. Med Sci Sports Exerc.

[15] Cavazzuti L, Merlo A, Orlandi F, Campanini I (2010). Delayed onset of electromyographic activity of vastus medialis obliquus relative to vastus lateralis in subjects with patellofemoral pain syndrome. Gait Posture.

[16] Chen HY, Chien CC, Wu SK, Liau JJ, Jan MH (2012). Electromechanical delay of the vastus medialis obliquus and vastus lateralis in individuals with patellofemoral pain syndrome. J Orthop Sports Phys Ther.

[17] Christou EA (2004). Patellar taping increases vastus medialis oblique activity in the presence of patellofemoral pain. J Electromyogr Kinesiol.

[18] Collins N, Crossley K, Beller E, Darnell R, McPoil T, Vicenzino B (2009). Foot orthoses and physiotherapy in the treatment of patellofemoral pain syndrome: randomised clinical trial. Br J Sports Med.

[19] Cowan SM, Bennell KL, Hodges PW, Crossley KM, McConnell J (2001). Delayed onset of electromyographic activity of vastus medialis obliquus relative to vastus lateralis in subjects with patellofemoral pain syndrome. Arch Phys Med Rehabil.

[20] Cowan SM, Bennell KL, Hodges PW (2002). Therapeutic patellar taping changes the timing of vasti muscle activation in people with patellofemoral pain syndrome. Clin J Sport Med.

[21] Crossley KM, Zhang WJ, Schache AG, Bryant A, Cowan SM (2011). Performance on the single-leg squat task indicates hip abductor muscle function. Am J Sports Med.

[22] D’hondt NE, Struijs PA, Kerkhoffs GM, Verheul C, Lysens R, Aufdemkampe G, Van Dijk CN (2002) Orthotic devices for treating patellofemoral pain syndrome. Cochrane Database Syst Rev (2):CD002267 10.1002/14651858.CD002267 12076444

[23] Domenech J, Sanchis-Alfonso V, López L, Espejo B (2013). Influence of kinesiophobia and catastrophizing on pain and disability in anterior knee pain patients. Knee Surg Sports Traumatol Arthrosc.

[24] Draper CE, Besier TF, Santos JM, Jennings F, Fredericson M, Gold GE, Beaupre GS, Delp SL (2009). Using real-time MRI to quantify altered joint kinematics in subjects with patellofemoral pain and to evaluate the effects of a patellar brace or sleeve on joint motion. J Orthop Res.

[25] Draper CE, Fredericson M, Gold GE, Besier TF, Delp SL, Beaupre GS, Quon A (2012). Patients with patellofemoral pain exhibit elevated bone metabolic activity at the patellofemoral joint. J Orthop Res.

[26] Dye SF (2005). The pathophysiology of patellofemoral pain: a tissue homeostasis perspective. Clin Orthop Relat Res.

[27] Ford KR, Myer GD, Hewett TE (2003). Valgus knee motion during landing in high school female and male basketball players. Med Sci Sports Exerc.

[28] Ford KR, Myer GD, Toms HE, Hewett TE (2005). Gender differences in the kinematics of unanticipated cutting in young athletes. Med Sci Sports.

[29] Fulkerson JP, Arendt EA (2000). Anterior knee pain in females. Clin Orthop Relat Res.

[30] Gilleard W, McConnell J, Parsons D (1998). The effect of patellar taping on the onset of vastus medialis obliquus and vastus lateralis muscle activity in persons with patellofemoral pain. Phys Ther.

[31] Gross MT, Foxworth JL (2003). The role of foot orthoses as an intervention for patellofemoral pain. J Orthop Sports Phys Ther.

[32] Harvie D, O’Leary T, Kumar S (2011). A systematic review of randomized controlled trials on exercise parameters in the treatment of patellofemoral pain: what works?. J Multidiscip Healthc.

[33] Heintjes E, Berger MY, Bierma-Zeinstra SM, Bernsen RM, Verhaar JA, Koes BW (2003). Exercise therapy for patellofemoral pain syndrome. Cochrane Database Syst Rev.

[34] Heintjes E, Berger MY, Bierma-Zeinstra SM, Bernsen RM, Verhaar JA, Koes BW (2004). Pharmacotherapy for patellofemoral pain syndrome. Cochrane Database Syst Rev.

[35] Hewett TE, Myer GD, Ford KR (2004). Decrease in neuromuscular control about the knee with maturation in female athletes. J Bone Joint Surg Am.

[36] Jensen R, Hystad T, Baerheim A (2005). Knee function and pain related to psychological variables in patients with long-term patellofemoral pain syndrome. J Orthop Sports Phys Ther.

[37] Jensen R, Hystad T, Kvale A, Baerheim A (2007). Quantitative sensory testing of patients with long lasting patellofemoral pain syndrome. Eur J Pain.

[38] Johnston LB, Gross MT (2004). Effects of foot orthoses on quality of life for individuals with patellofemoral pain syndrome. J Orthop Sports Phys Ther.

[39] Kaya D, Doral MN (2012). Is there any relationship between Q-angle and lower extremity malalignment?. Acta Orthop Traumatol Turc.

[40] Kettunen JA, Harilainen A, Sandelin J, Schlenzka D, Hietaniemi K, Seitsalo S, Malmivaara A, Kujala UM (2007). Knee arthroscopy and exercise versus exercise only for chronic patellofemoral pain syndrome: a randomized controlled trial. BMC Med.

[41] Lankhorst NE, Bierma-Zeinstra SM, van Middelkoop M (2013). Factors associated with patellofemoral pain syndrome: a systematic review. Br J Sports Med.

[42] Levinger P, Gilleard W (2007). Tibia and rearfoot motion and ground reaction forces in subjects with patellofemoral pain syndrome during walking. Gait Posture.

[43] MacIntyre NJ, Hill NA, Fellows RA, Ellis RE, Wilson DR (2006). Patellofemoral joint kinematics in individuals with and without patellofemoral pain syndrome. J Bone Joint Surg Am.

[44] Mølgaard M (2011). Patellofemoral pain syndrome and its association with hip, ankle, and foot function in 16- to 18-year-old high school students: a single-blind case-control study. J Am Podiatr Med Assoc.

[45] Myer GD, Ford KR, Barber Foss KD, Goodman A, Ceasar A, Rauh MJ, Divine JG, Hewett TE (2010). The incidence and potential pathomechanics of patellofemoral pain in female athletes. Clin Biomech (Bristol, Avon).

[46] Padua DA, Marshall SW, Beutler AI, Demaio M, Boden BP, Yu B, Garrett WE (2005). Predictors of knee valgus angle during a jump-landing task. Med Sci Sports Exerc.

[47] Patil S, White L, Jones A, Hui AC (2010). Idiopathic anterior knee pain in the young. A prospective controlled trial. Acta Orthop Belg.

[48] Patil S, Dixon J, White LC, Jones AP, Hui AC (2011). An electromyographic exploratory study comparing the difference in the onset of hamstring and quadriceps contraction in patients with anterior knee pain. Knee.

[49] Pattyn E, Verdonk P, Steyaert A, Vanden Bossche L, Van den Broecke W, Thijs Y, Witvrouw E (2011). Vastus medialis obliquus atrophy: does it exist in patellofemoral pain syndrome?. Am J Sports Med.

[50] Pal S, Draper CE, Fredericson M, Gold GE, Delp SL, Beaupre GS, Besier TF (2011). Patellar maltracking correlates with vastus medialis activation delay in patellofemoral pain patients. Am J Sports Med.

[51] Park SK, Stefanyshyn DJ (2011). Greater Q angle may not be a risk factor of patellofemoral pain syndrome. Clin Biomech (Bristol, Avon).

[52] Petersen W, Ellermann A, Liebau C, Brüggemann GP, Best R, Gösele-Koppenburg A, Semsch H, Albasini A, Rembitzki I (2010). Das patellofemorale schmerzsyndrom. Orthopädische Praxis.

[53] Pfeiffer RP, DeBeliso M, Shea KG, Kelley L, Irmischer B, Harris C (2004). Kinematic MRI assessment of McConnell taping before and after exercise. Am J Sports Med.

[54] Piva SR, Fitzgerald GK, Wisniewski S, Delitto A (2009). Predictors of pain and function outcome after rehabilitation in patients with patellofemoral pain syndrome. J Rehabil Med.

[55] Piva SR, Fitzgerald GK, Irrgang JJ, Fritz JM, Wisniewski S, McGinty GT, Childs JD, Domenech MA, Jones S, Delitto A (2009). Associates of physical function and pain in patients with patellofemoral pain syndrome. Arch Phys Med Rehabil.

[56] Powers CM, Ward SR, Chen YJ, Chan LD, Terk MR (2004). Effect of bracing on patellofemoral joint stress while ascending and descending stairs. Clin J Sport Med.

[57] Prins MR, van der Wurff P (2009). Females with patellofemoral pain syndrome have weak hip muscles: a systematic review. Aust J Physiother.

[58] Rathleff MS, Roos EM, Olesen JL, Rasmussen S, Arendt-Nielsen L (2013). Lower mechanical pressure pain thresholds in female adolescents with patellofemoral pain syndrome. J Orthop Sports Phys Ther.

[59] Rauh MJ, Koepsell TD, Rivara FP, Rice SG, Margherita AJ (2007). Quadriceps angle and risk of injury among high school cross-country runners. J Orthop Sports Phys Ther.

[60] Robinson RL, Nee RJ (2007). Analysis of hip strength in females seeking physical therapy treatment for unilateral patellofemoral pain syndrome. J Orthop Sports Phys Ther.

[61] Sanchis-Alfonso V, Roselló-Sastre E (2000). Immunohistochemical analysis for neural markers of the lateral retinaculum in patients with isolated symptomatic patellofemoral malalignment. A neuroanatomic basis for anterior knee pain in the active young patient. Am J Sports Med.

[62] Sanchis-Alfonso V (2008) Patellofemoral pain. Orthopade 37(9):835-6, 838–840 10.1007/s00132-008-1289-2 18682915

[63] Souza RB, Draper CE, Fredericson M, Powers CM (2010). Femur rotation and patellofemoral joint kinematics: a weight-bearing magnetic resonance imaging analysis. J Orthop Sports Phys Ther.

[64] Thomas MJ, Wood L, Selfe J, Peat G (2010). Anterior knee pain in younger adults as a precursor to subsequent patellofemoral osteoarthritis: a systematic review. BMC Musculoskelet Disord.

[65] Thomeé P, Thomeé R, Karlsson J (2002). Patellofemoral pain syndrome: pain, coping strategies and degree of well-being. Scand J Med Sci Sports.

[66] Tsuji T, Matsuyama Y, Goto M, Yimin Y, Sato K, Hasegawa Y, Ishiguro N (2002). Knee-spine syndrome: correlation between sacral inclination and patellofemoral joint pain. J Orthop Sci.

[67] Utting MR, Davies G, Newman JH (2005). Is anterior knee pain a predisposing factor to patellofemoral osteoarthritis?. Knee.

[68] Vicenzo B, Collins N, Crossley K, Beller E, Darnell R, McPoil T (2008). Foot orthoses and physiotherapy in the treatment of patellofemoral pain syndrome: a randomised clinical trial. BMC Musculoskelet Disord.

[69] Vicenzo B, Collins N, Cleland J, McPoil T (2010). A clinical prediction rule for identifying patients with patellofemoral pain who are likely to benefit from foot orthoses: a preliminary determination. Br J Sports Med.

[70] Warden SJ, Hinman RS, Watson MA, Avin KG, Bialocerkowski AE, Crossley KM (2008). Patellar taping and bracing for the treatment of chronic knee pain: a systematic review and meta-analysis. Arthr Rheum.

[71] White LC, Dolphin P, Dixon J (2009). Hamstring length in patellofemoral pain syndrome. Physiotherapy.

[72] Wilson NA, Press JM, Koh JL, Hendrix RW, Zhang LQ (2009). In vivo noninvasive evaluation of abnormal patellar tracking during squatting in patients with patellofemoral pain. J Bone Joint Surg Am..

[73] Wiener-Ogilvie S, Jones RB (2004). A randomised trial of exercise therapy and foot orthoses as treatment for knee pain in primary care. Br J Podiatry.

[74] Witvrouw E, Lysens R, Bellemans J, Cambier D, Vanderstraeten G (2000). Intrinsic risk factors for the development of anterior knee pain in an athletic population. A two-year prospective study. Am J Sports Med.

[75] Wojtys EM, Beaman DN, Glover RA, Janda D (1990). Innervation of the human knee joint by substance-P fibers. Arthroscopy.

[76] Wu CC, Shih CH (2004). The influence of iliotibial tract on patellar tracking. Orthopedics.

